# Combined Anatomical Arthroscopic Posterior Cruciate Ligament and Posterolateral Corner Reconstruction Using a Knotless Anchor: A Simplified Approach

**DOI:** 10.1016/j.eats.2024.103310

**Published:** 2024-11-25

**Authors:** Ali Alayane, David Zhu, Dany Mouarbes, Lea Estivals, Etienne Cavagnaic

**Affiliations:** a Clinique Universitaire du Sport, Centre Hospitalier Universitaire de Toulouse (CHU), Toulouse, France; b Centre Hospitalier de Perpignan (CHP), Perpignan, France; c Centre Chirurgical Emile Gallé, Nancy, France; d Service de Chirurgie Orthopédique Traumatologique et Arthroscopique, CHRU Nancy, Nancy, France

## Abstract

Posterior cruciate ligament injuries are commonly associated with the knee joint posterolateral corner injury. Many open surgical techniques have been described in the literature to restore knee joint stability, including reconstruction of the posterior cruciate ligament and the posterolateral corner. However, few articles discussed the arthroscopic reconstruction of such multiligament knee injury. In this Technical Note, we describe an entirely simplified arthroscopic technique for anatomical reconstruction of the knee posterior cruciate ligament and posterolateral corner using the transseptal approach and a knotless anchor for posterolateral corner graft femoral fixation.

Posterolateral corner (PLC) is a complex anatomical structure composed of the lateral collateral ligament, the popliteus muscle and its tendon, the popliteofibular ligament, the posterolateral capsule, the fabellofibular ligament, and the arcuate ligament.^1^ The main function of the PLC is to resist the lateral joint opening and the posterior subluxation of the lateral tibial plateau during tibial rotation, thereby preventing knee hyperextension and varus recurvatum.^2^ Isolated PLC injury is uncommon and its usually associated with posterior cruciate ligament (PCL) injury.^3^ Grade 3 posterior knee laxity, according to the International Knee Documentation Committee classification,^4^ is considered a clinical indicator of associated PCL and PLC injuries.^5^ PLC deficiency results in varus malalignment, potentially inducing pain in the medial tibiofemoral compartment secondary to osteoarthritis. In addition, PLC deficiency is considered a major risk factor for PCL reconstruction failure.^6^

Multiple surgical techniques have been described in the literature for anatomical PLC reconstruction, aiming to restore knee joint stability and kinematics.^7^^,^^8^ However, these procedures typically require extensive surgical approaches involving identification and protection of the peroneal nerve. Chronic PLC reconstruction becomes more challenging because of the presence of significant soft-tissue adhesions and the alteration in the anatomy of the lateral knee structures. Recently, there is a growing interest in the use of arthroscopy to perform PLC reconstruction.^9^, ^10^, ^11^ Combined PLC and PCL reconstruction is considered a challenging procedure requiring advanced arthroscopic surgical skills because of the complexity of the posterolateral knee structures and the proximity of the popliteal neurovascular structures.^12^

In this article, we describe an arthroscopic technique for PLC and PCL reconstruction using the transseptal approach. The technique focuses on the accurate positioning of the PLC graft within the popliteus sulcus footprint. In addition, PLC femoral fixation is simplified by using a knotless soft anchor (Video 1). Pearls and pitfalls are described in Table 1. Advantages and disadvantages are presented in Table 2.

**Table 1:** Surgical Steps: Pearls and Pitfalls

Surgical Steps	Pearls	Pitfalls
Transeptal approach	Use of a needle for accurate creation of PM and PL portals under direct skin trans illumination. Careful separation of the posterior capsule from the PCL footprint ensures the preservation of PCL remnants for a better biological healing.	Careful debridement of the septum using the shaver with the cutting edge always facing the bone to avoid posterior neurovascular injury.
Lateral gutter preparation and knotless soft anchor insertion	Creation of the LEP under direct skin transillumination using a 21-gauge needle to accurately identify the lateral epicondylar footprint for the PLC insertion.	Lateral gutter soft-tissue debridement is essential to avoid the knotless anchor malposition. The anchor should be inserted in the same direction of the drilled lateral epicondylar tunnel to avoid anchor breakage.
PCL and PLC tibial tunnel creation	Arthroscopic visualization of tunnels creation is achieved by inserting the arthroscope through the PM portal to ensure proper tunnel position.	The PLC footprint should be well prepared by removing soft tissues to visualize the tibial sulcus, thereby preventing PLC graft malposition.
PCL and PLC graft passage and femoral fixation	The endobutton is pulled through the PCL femoral tunnel under direct arthroscopic visualization via the AM portal to ensure adequate passage and tightening on the medial femoral epicondyle before pulling the PCL graft into the femoral tunnel. The PLC graft is visualized in the lateral gutter to ensure accurate positioning and fixation at the lateral epicondyle	The PLC graft should be passed anterior to the lateral collateral ligament, avoiding any soft-tissue interposition between the PLC graft and the femoral condyle.
PCL and PLC grafts tibial fixation	Holding the K-wire while inserting the screws for tibial fixation is recommended to avoid iatrogenic posterior neurovascular injury. Double PLC tibial fixation is achieved using 2 interference screws. The first screw reaches the posterior tibial cortex under direct arthroscopic visualization, while the second screw is flush to the anterior tibial cortex ensuring accurate cortical bone fixation of the PCL graft It’s recommended to use arthroscopic visualization of both grafts through the PM portal during the fixation of the tibial screws to ensure adequate grafts tensioning.	PLC and PCL graft fixation should be done at 90° of knee flexion with the knee in a reduced position and internally rotated to avoid residual knee laxity.

LEP, lateral epicondylar portal; PCL, posterior cruciate ligament; PL, posterolateral; PLC, posterolateral corner; PM, posteromedial.

**Table 2:** Advantages and Disadvantages

Advantages	Disadvantages
Arthroscopic anatomic PCL and PLC reconstruction. Mini-invasive PLC reconstruction; no need to identify and protect the peroneal nerve Better visualization of the PCL and PLC tibial tunnel footprint.	The transseptal approach requires a significant learning curve. Risk of popliteal neurovascular injury when performing the tibial tunnels. Risk of knotless soft anchor malposition if the lateral gutter not adequately prepared.
PCL remnants and meniscofemoral ligament preservation improving graft healing.	
Simple and solid lateral condyle PLC graft fixation can be achieved using a knotless soft anchor through the LEP after lateral gutter exploration. It avoids the need of femoral tunnel creation and screw fixation complications. Preservation of the cancellous bone in the distal femur for potential future revisions avoiding the risk of tunnels conversion.	

LEP, lateral epicondylar portal; PCL, posterior cruciate ligament; PL, posterolateral; PLC, posterolateral corner; PM, posteromedial.

## Surgical Technique

### Patient Setup

Under general anesthesia, the patient is placed in supine position and a tourniquet is applied on the operative thigh. The knee is then assessed for full range of motion and confirmed to be stable on the foot roll at 90° of flexion. Posterior knee instability and increased tibial external rotation are confirmed by performing the posterolateral drawer test preoperatively (Video 1).

### PCL and PLC Graft Preparation

**Fig 1:**
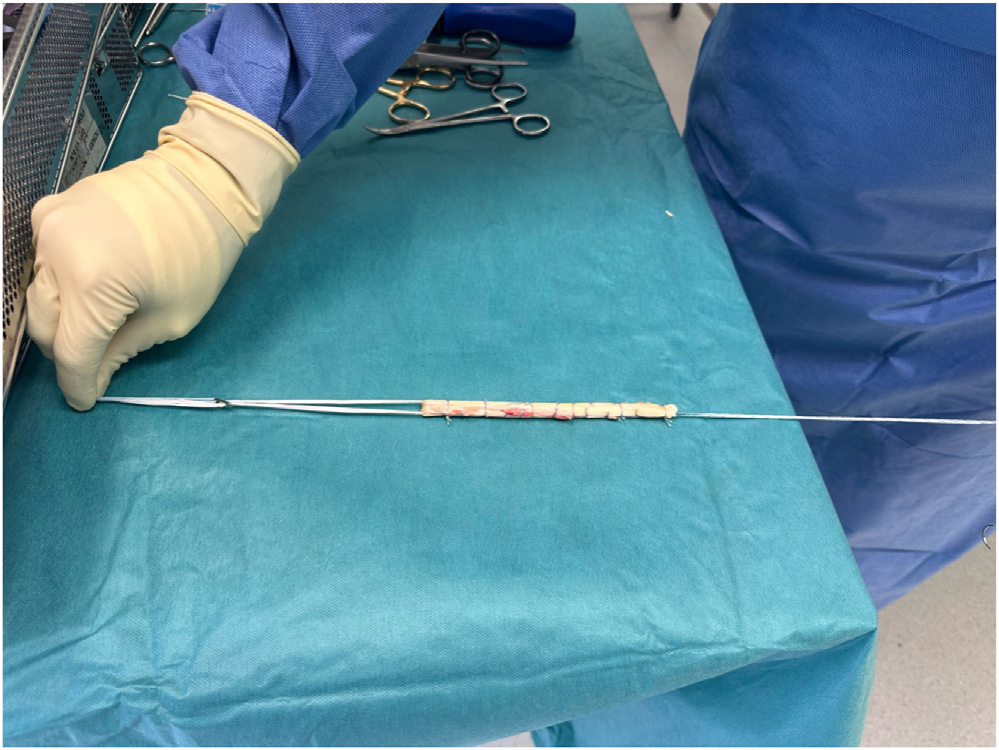
Intraoperative photograph showing the prepared posterior cruciate ligament (PCL) graft using a tibialis posterior tendon allograft, sutured to itself and looped with an adjustable TightRope to obtain a 12-cm PCL graft measuring 9 mm in diameter.

A fresh frozen and nonirradiated tibialis posterior allograft is soaked in the antibiotic rifampicin for 20 minutes before its use as a preventive measure to minimize the risk of infection. Subsequently, a 12-cm PCL graft measuring 9 mm in diameter is prepared and looped with a TightRope RT implant (Arthrex, Naples, FL) (Fig 1).

**Fig 2:**
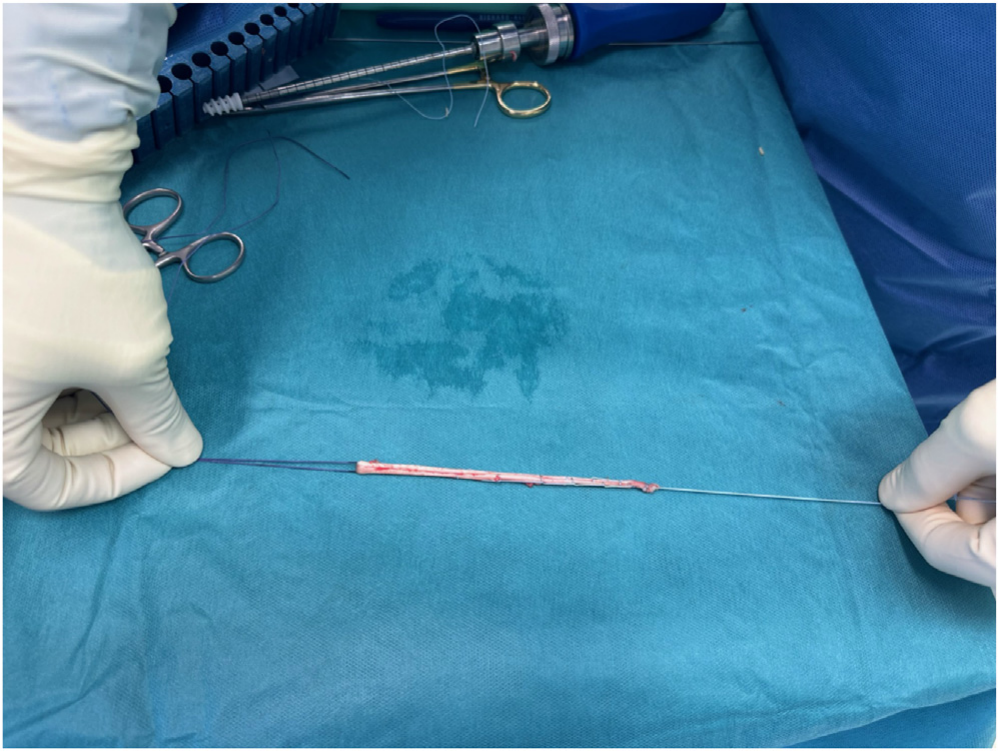
Intraoperative photograph showing the prepared posterolateral corner graft using a gracilis tendon autograft, doubled and sutured to itself and looped around 2.0 loop suture.

The gracilis tendon is harvested and then a double-stranded 7-mm diameter PLC graft is prepared by passing a No. 2 FiberLoop with a straight needle (Arthrex) through both ends of the graft and looped with a 2.0 VICRYL traction suture (Fig 2). The PLC graft is subsequently soaked in a vancomycin solution before implantation.

### Transseptal Approach and Creation of Posterior Portals

**Fig 3:**
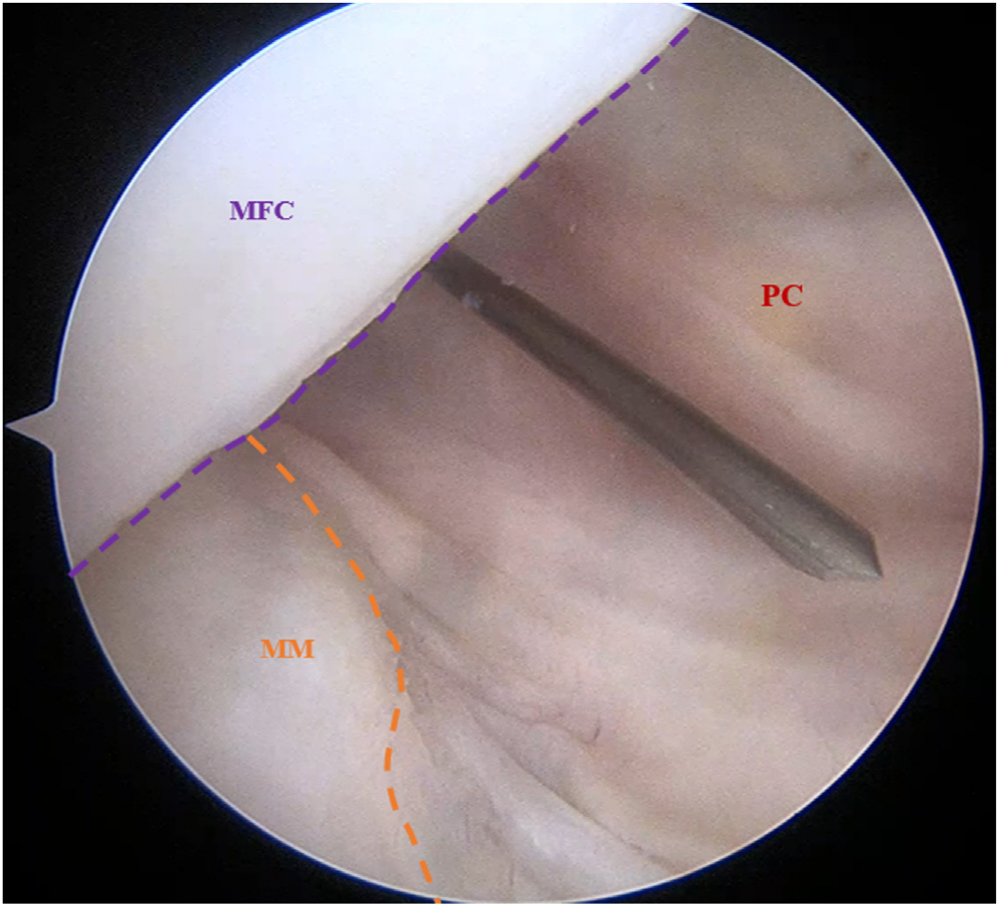
Arthroscopic view of the left knee in 90° of flexion with the arthroscope in the posteromedial knee compartment through the transnotch approach. Shown is PM portal creation using an 18-gauge needle. (MFC, medial femoral condyle; MM, medial meniscus; PC, posterior capsule; PM, posteromedial.)

**Fig 4:**
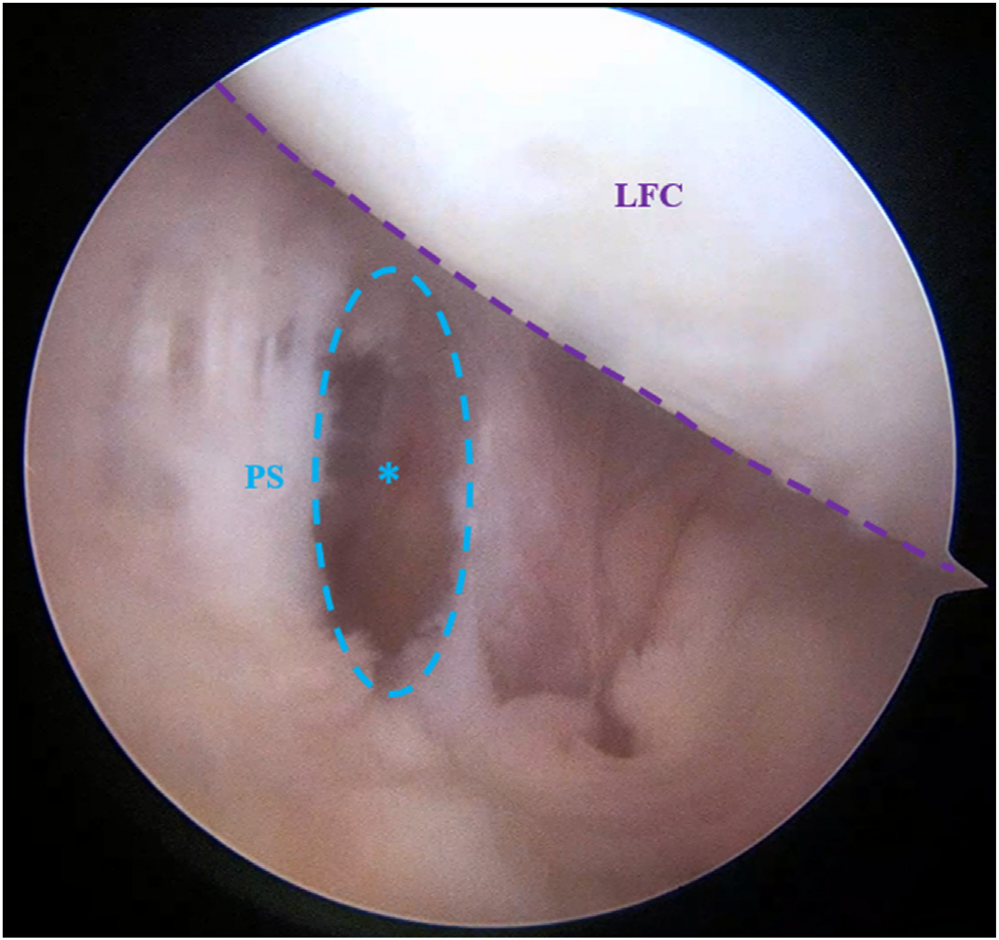
Arthroscopic view of the left knee in 90° of flexion with the arthroscope inserted through the PM portal showing the opening of the posterior septum to create the transseptal approach. The shaver is inserted through the PM portal under direct arthroscopic visualization via the transnotch approach. The cutting surface of the shaver is consistently oriented toward the bone to prevent potential popliteal vascular injury during posterior septal opening creation. ∗Posterior septum opening. (LFC, lateral femoral condyle; PM, posteromedial; PS, posterior septum.)

**Fig 5:**
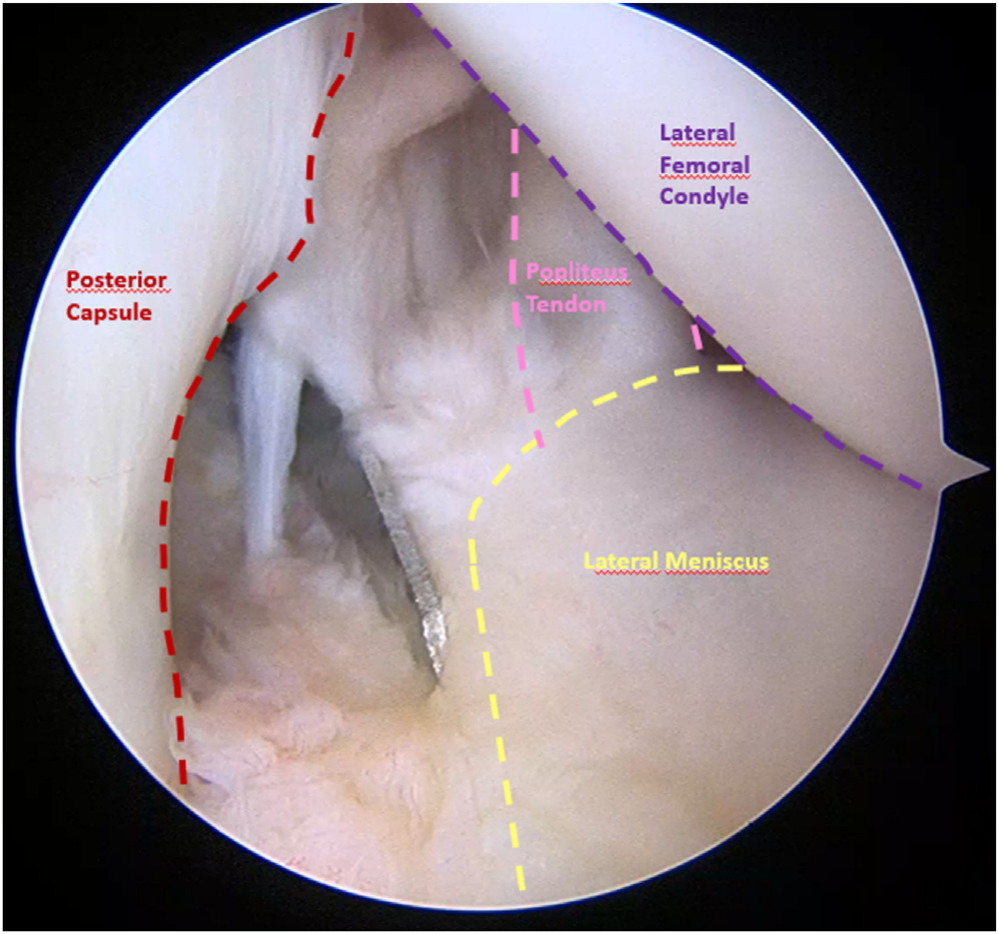
Arthroscopic view of the left knee in 90° of flexion, showing the PL compartment using the transseptal approach with the scope inserted through the PM portal. The PL portal is created using an 18-gauge needle under direct skin transillumination. The PL capsule is then separated from the posterior horn of the lateral meniscus with a No. 11 blade scalpel to prepare the PLC footprint at the popliteus sulcus. (PL, posterolateral; PLC, posterolateral corner; PM, posteromedial.)

Using a 30° arthroscope, a diagnostic arthroscopy is performed through standard anterolateral and anteromedial (AM) portals to confirm posterior knee instability. Using the transnotch approach, the posteromedial (PM) portal is established (Fig 3). Then under arthroscopic visualization, the debridement of the posterior septum is performed using the shaver through PM portal, ensuring constant orientation of the cutting surface toward the bone to prevent potential popliteal vascular injury. The scope is inserted through the PM portal, and advanced through the septal opening (Fig 4), reaching the posterolateral knee compartment to create the posterolateral (PL) portal under direct visualization using skin transillumination (Fig 5).

**Fig 6:**
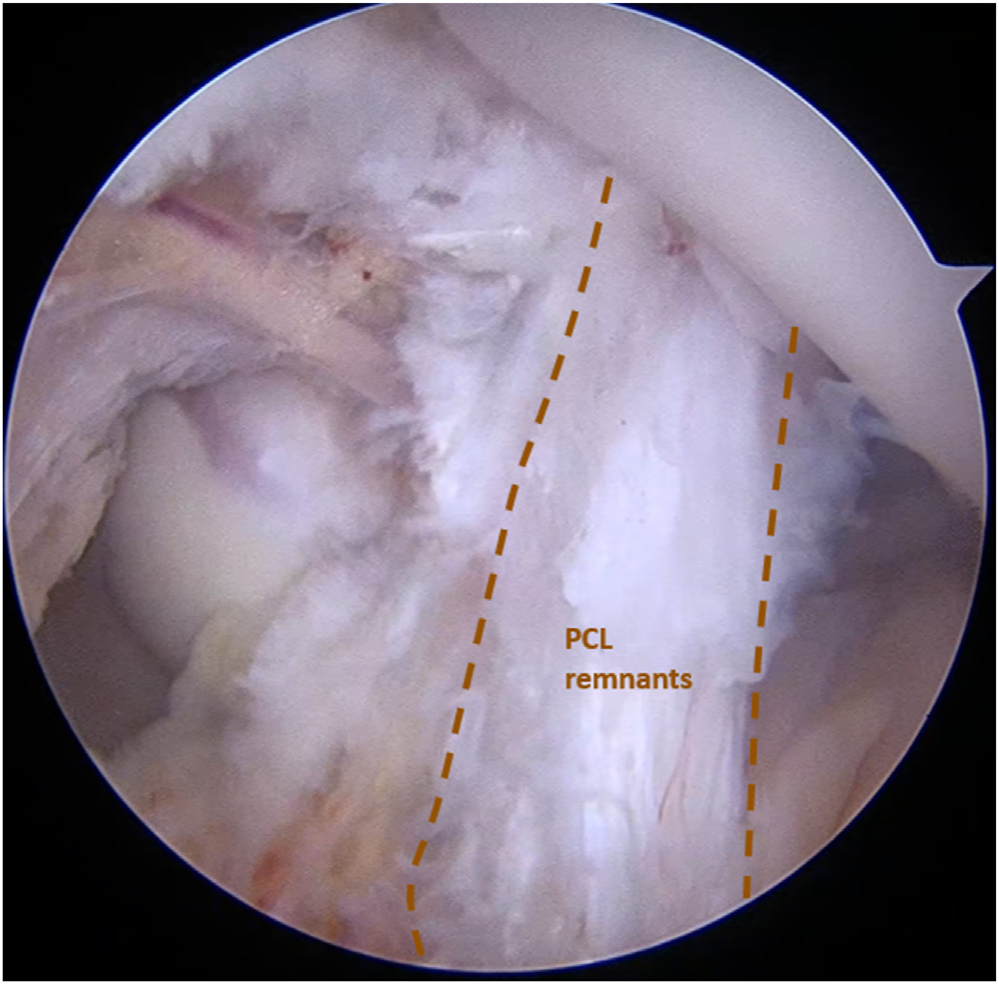
Arthroscopic view of the left knee in 90° of flexion with the arthroscope inserted through the posteromedial portal showing the PCL remnants after separation of the posterior capsule using an electrocoagulation cautery. (PCL, posterior cruciate ligament.)

**Fig 7:**
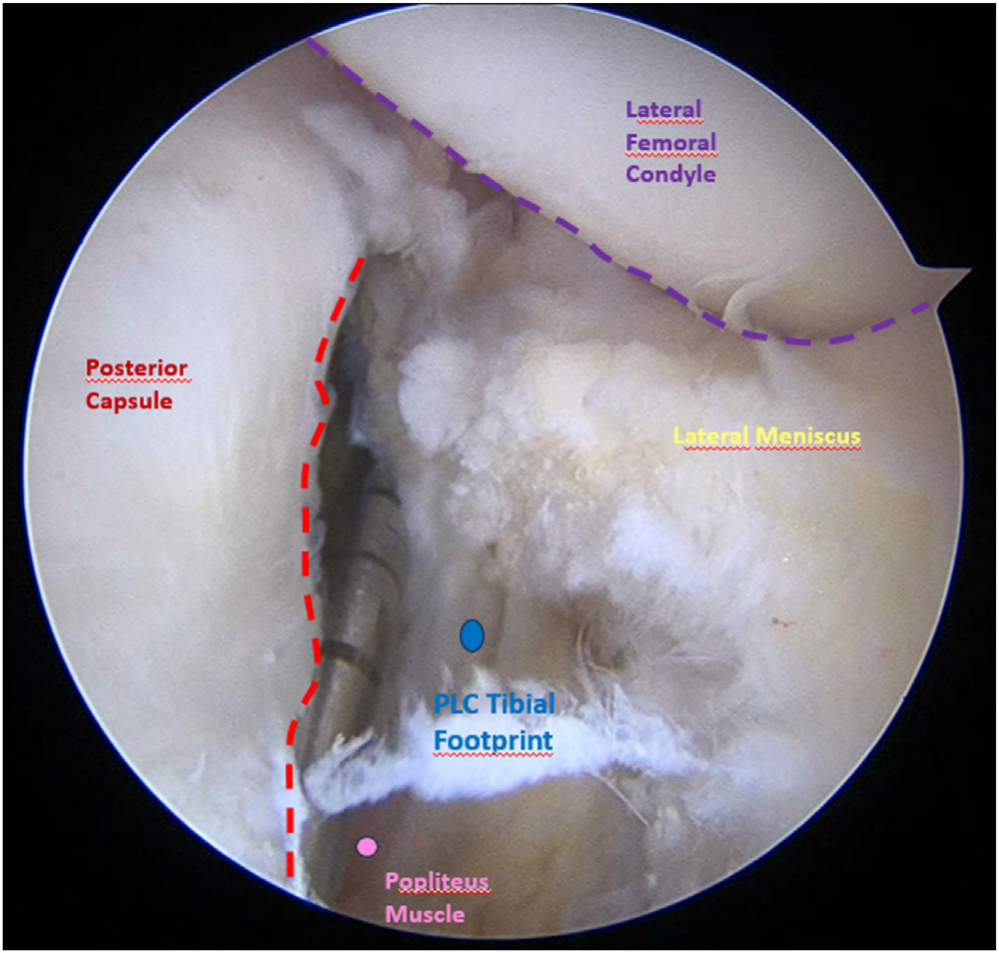
Arthroscopic view of the left knee in 90° of flexion. Shown is the use of the transseptal approach with the scope inserted through the PM portal, revealing the posterolateral compartment structures and the prepared PLC footprint at the popliteus sulcus. Soft tissues are cleaned using a shaver through PL portal to define the anatomical PLC footprint. (PL, posterolateral; PLC, posterolateral corner; PM, posteromedial.)

A radiofrequency device is introduced through the PL portal to first separate the septum from the capsule allowing visualization of the PCL tibial footprint (Fig 6). Subsequently, a meticulous dissection is performed to separate the lateral posterior capsule from the posterior horn of the lateral meniscus, exposing the PLC footprint at the popliteal sulcus (Fig 7).

### PCL Femoral Tunnel Creation and Lateral Gutter Preparation

**Fig 8:**
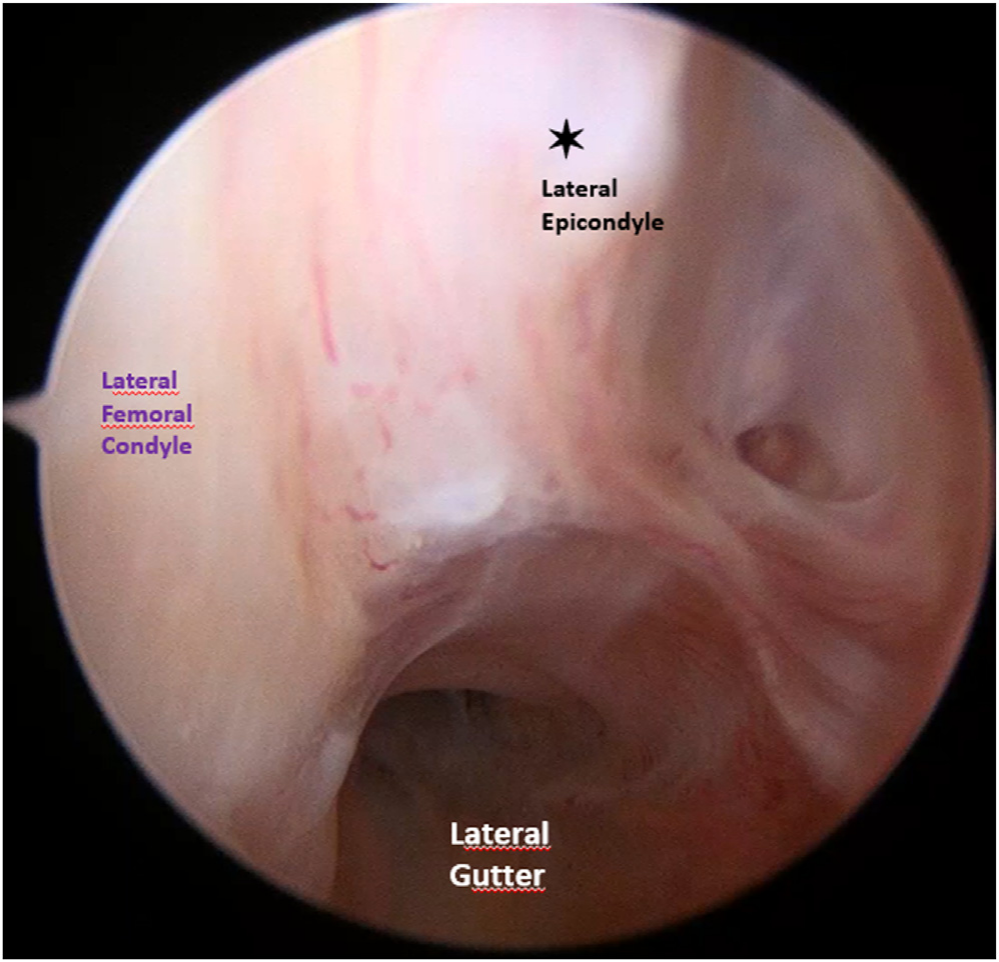
Arthroscopic view of the left knee, with the arthroscope inserted through the anterolateral portal, showing exploration of the lateral gutter and identification of the lateral epicondyle with the knee flexed at 90°.

**Fig 9:**
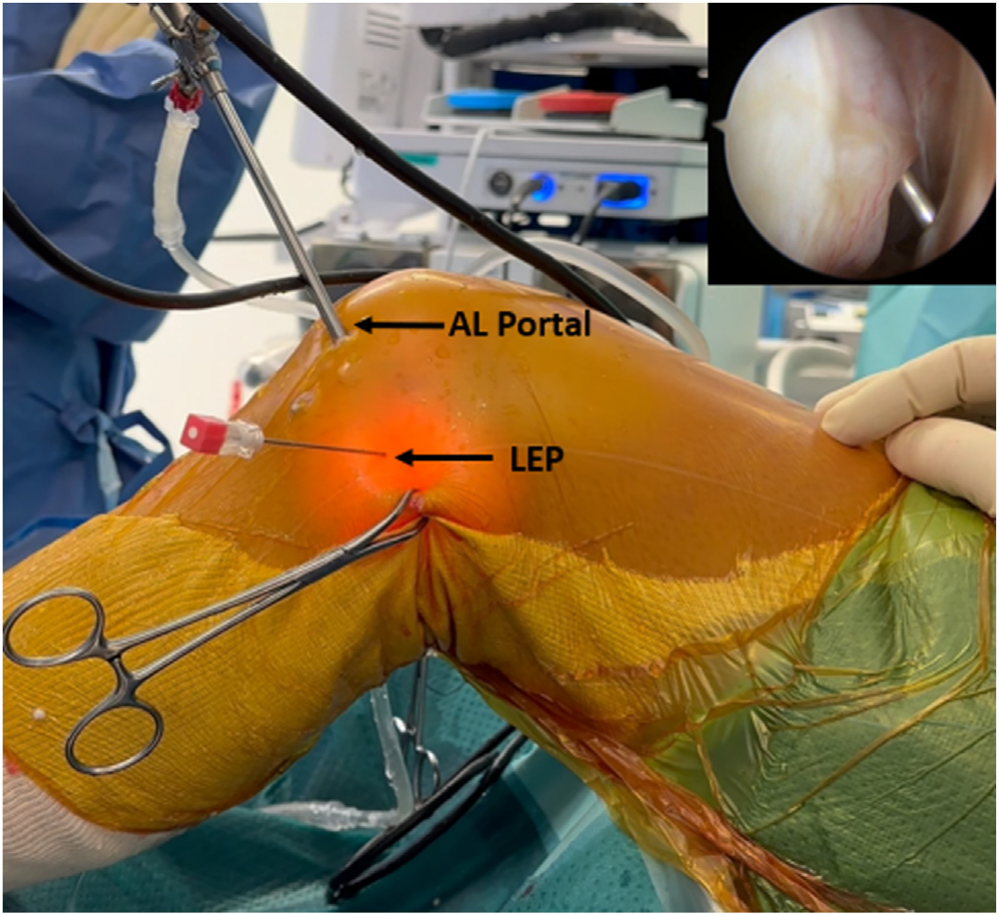
Intraoperative photograph of the left knee in 90° of flexion. Shown is the creation of the lateral epicondylar portal (LEP) with direct arthroscopic visualization of the lateral gutter through the anterolateral portal. A 21-gauge needle is used to accurately identify the position of the knotless anchor at the lateral epicondyle. The soft tissue around the lateral epicondyle is then completely cleared using a shaver to prepare for anchor insertion.

**Fig 10:**
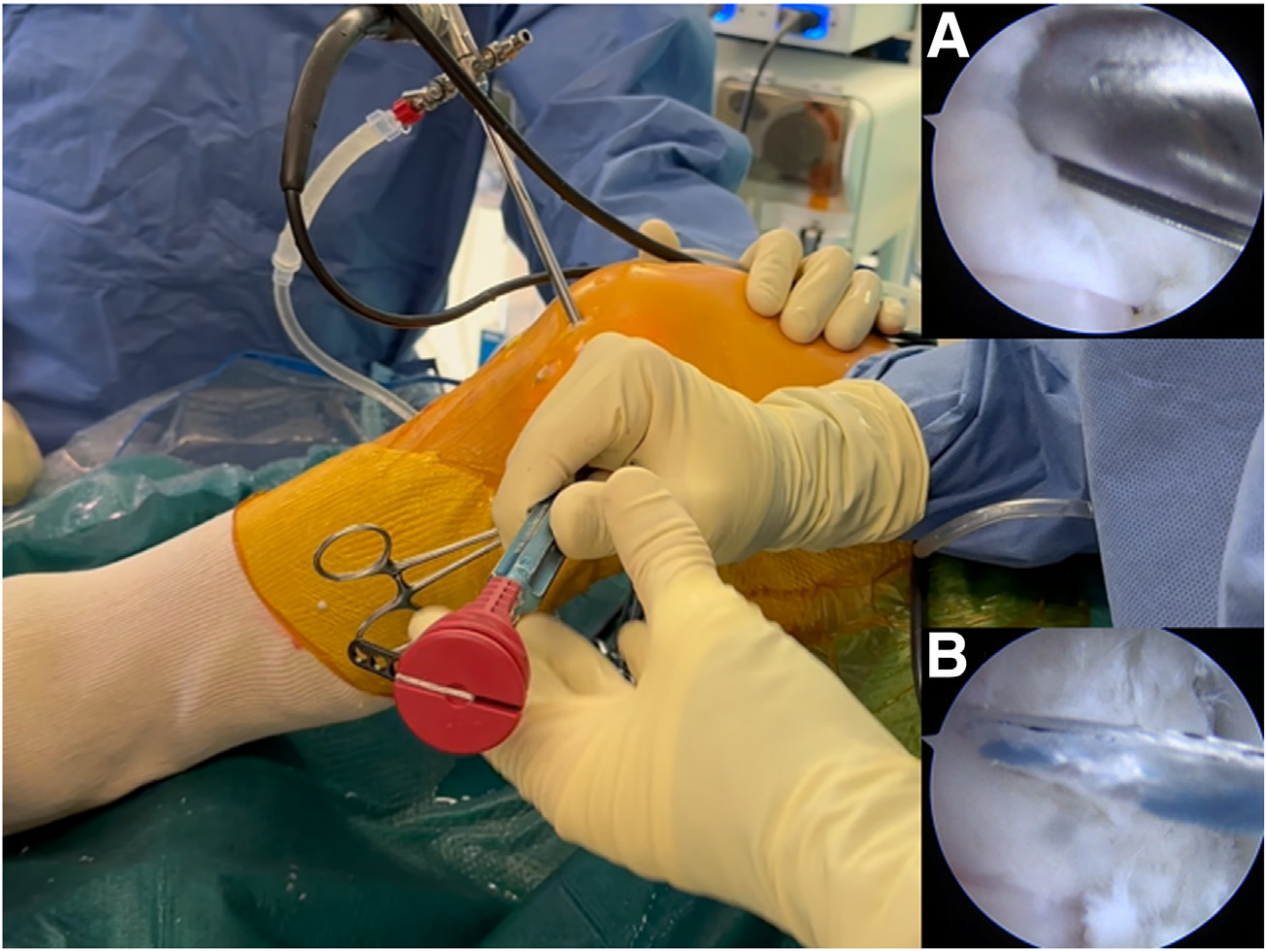
Intraoperative photograph of the left knee in 90° of flexion. (A) Shown is the insertion of the knotless soft anchor though the lateral epicondylar portal with direct arthroscopic visualization of the lateral gutter through the anterolateral portal. (B) Traction is applied to confirm satisfactory anchor fixation.

The PCL femoral tunnel is performed by inserting a guidewire through the PCL footprint, which is located 10 mm posterior to the medial femoral condyle cartilage and directed proximal and slightly posterior to the lateral femoral epicondyle, then it’s overdrilled from inside to outside using a 9-mm drill bit to create a 20-mm long femoral tunnel then a loop suture is positioned in the tunnel. Subsequently, the arthroscope is inserted into the lateral gutter (Fig 8), and under arthroscopic guidance, a 21-gauge needle is used to accurately identify the lateral epicondylar portal (LEP) (Fig 9). The soft tissue around the lateral epicondyle is then completely cleared using a shaver to prepare for anchor insertion. A 1.8-mm knotless FiberTak Soft Anchor (Arthrex) is then inserted into the lateral femoral cortex through a predrilled 2.6 × 35 mm and tested by applying traction to confirm satisfactory fixation (Fig 10).

### PCL and PLC Tibial Tunnel Creation

After performing accurate exposure of the PCL remnants and under direct visualization of the arthroscope through the PM portal, the hook of the PCL guide is introduced through the AM portal and positioned at the PCL tibial footprint at 10 mm downward from the articular surface. A guidewire is then inserted through the medial AM tibial cortex at the level of the hamstring’s insertion to the PL tibial footprint and its overdrilled with a 9-mm drill bit, then a FiberStick suture is inserted into the tibial tunnel and retrieved from the AM portal.

The PCL tibial guide is reinserted through the PL portal and positioned at the popliteus sulcus footprint under direct arthroscopic visualization though the PM portal. Subsequently, a guidewire is inserted over the Gerdy tubercule and over drilled with 7-mm reamer. A loop suture is passed through the PCL tibial tunnel and retrieved from the LEP.

### PCL and PLC Graft Passage and Femoral Fixation

While maintaining the knee at 90° of flexion, the PCL graft is passed through the tibial tunnel using the fiber loop suture and the endobutton is pulled into the femoral tunnel under direct arthroscopic control through and AM portal. Then the PCL allograft is gradually pulled up to fill the femoral tunnel. The endobutton is positioned and tightened on the medial femoral condyle. Subsequently, the PLC graft is pulled through the tibial tunnel using the loop suture under direct arthroscopic visualization through the PM portal and its looped end of is pulled out by the fiber loop suture from the LEP.

### PCL and PLC Graft Tibial Fixation

A double fixation of the PCL allograft into the tibial tunnel is performed using 2 interference screws. The first 9-mm × 25-mm interference screw is inserted until the posterior cortex of the tibia and the second 10-mm × 25-mm interference screw is inserted flush to the anterior entry of the tibial tunnel while maintaining the knee in reduced position at 90°of flexion. Subsequently, the PLC graft is fixed with a 7-mm bioabsorbable screw with the knee held in internal rotation. It’s crucial to hold the k-wire during screws fixation to avoid any iatrogenic injury to the posterior neurovascular structures. Postoperative posterior and PL drawer testing are performed to ensure adequate knee stability.

### Postoperative Protocol

The postoperative protocol includes 6 weeks of PCL knee immobilization with partial weight-bearing while keeping the knee in full extension. Early passive range of motion exercises are allowed in the prone position, with flexion greater than 90° avoided. Total weight-bearing is allowed 6 weeks postoperatively. Then, the patient is allowed to return to sports, starting with nonpivoting activities at 6 months, followed by pivoting sports 9 months postoperatively.

## Discussion

PLC knee injuries are commonly associated with PCL injuries. Numerous open surgical techniques have been described in the literature for anatomical PLC reconstruction.^13^^,^^14^ The aim of these surgical procedures is to restore anteroposterior and rotational knee stability.^15^ However, these procedures often require extensive open surgical dissection to achieve anatomical reconstruction of the complex PLC structures, necessitating the identification and the protection of the peroneal nerve.^16^^,^^17^ Arthroscopic PLC and PCL reconstruction is gaining popularity as a safe and minimally invasive procedure for PL knee stabilization.

This article provides a simplified arthroscopic technique for PCL and PLC reconstruction. The transseptal approach allows better visualization of the PM and the PL knee compartment, reducing the risk of PLC and PCL tibial tunnel malposition. It also preserves the PCL remnants and the meniscofemoral ligament, which are crucial for improved graft healing and knee stabilization.^18^ In addition, the lateral border of the popliteus sulcus is considered an accurate and anatomical footprint for popliteus complex reconstruction on the basis of the cadaveric study done by Germon et al.^10^ Furthermore, PLC femoral fixation using the knotless soft anchor is a simple and efficient technique preventing the need of femoral tunnel creation and the complications related to the screw fixation. This technique also preserves cancellous bone in the distal femur for potential future revisions avoiding the risk of tunnels convergence.^19^

The main risk associated with this technique is posterior neurovascular injury, which is prevented by creating a good posterior space for working and holding k wires during tunnels reaming and screw fixation. Moreover, preparing the lateral gutter before anchor insertion is essential to avoid anchor malposition or breakage.

In our perspective, we believe that our technique offers a safe and simplified approach for arthroscopic PCL and PLC reconstruction. The authors encourage the use the transseptal approach for better visualization of the posterior tibial tunnel’s footprint. In addition, using a knotless soft anchor through LEP for PLC fixation on the lateral femoral condyle, after lateral gutter preparation, is a safe and efficient procedure.

## Disclosures

The authors declare the following financial interests/personal relationships which may be considered as potential competing interests: E.C. reports consulting or advisory with Arthrex. All other authors (A.A., D.Z., D.M., L.E.) declare that they have no known competing financial interests or personal relationships that could have appeared to influence the work reported in this paper.

## Supplementary Data

Video 1 This instructional video demonstrates a simplified arthroscopic technique for the (left knee) anatomical posterior cruciate ligament and posterolateral corner (PLC) reconstruction. The (under general anesthesia the patient is placed in supine position) first stage involves the creation of posteromedial (PM) and posterolateral portals using the transseptal approach. The second stage involves arthroscopic lateral femoral gutter exploration and lateral epicondylar insertion of the knotless soft anchor for PLC graft femoral fixation. Subsequently, posterior cruciate ligament and PLC tibial tunnels are created under direct arthroscopic visualization through the PM portal. Keys steps include transseptal approach, lateral gutter exploration and preparation for knotless soft anchor insertion, and anatomical footprint creation of tibial tunnels under arthroscopic visualization through the PM portal. The goal of this procedure is to achieve anatomical reconstruction of the PLC using a simplified knotless anchor fixation, avoiding additional femoral tunnel creation and preserving the cancellous bone in the distal femur.
