# Erratum

**DOI:** 10.1016/j.eats.2025.103987

**Published:** 2025-10-22

In the article, "Combined Anatomical Arthroscopic Posterior Cruciate Ligament and Posterolateral Corner Reconstruction Using a Knotless Anchor: A Simplified Approach”, published in the April 2025 issue (*Arthroscopy Techniques* 2025;14:103310), Etienne Cavaignac’s name was misspelled in the byline.

